# Closer to the Heart: Cardiac Muscle Aerobic Capacity Correlates With Intraspecific Variation in Sprint Performance Rather Than Androgen Levels in the Neotropical Lizard *Tropidurus catalanensis*

**DOI:** 10.1002/jez.70040

**Published:** 2025-09-22

**Authors:** Willian Souza Lima, Danilo Giacometti, Paul J. Schaeffer, José Eduardo de Carvalho

**Affiliations:** ^1^ Department of Ecology and Evolutionary Biology Universidade Federal de São Paulo São Paulo Brazil; ^2^ Department of Biological Sciences Brock University St. Catharines Ontario Canada; ^3^ Department of Physiology Universidade de São Paulo São Paulo Brazil; ^4^ Department of Biology Miami University Oxford Ohio USA

**Keywords:** citrate synthase, ectothermic, energy metabolism, heart metabolism, lactate dehydrogenase, morphology, muscle performance, sprint speed, testosterone, Tropiduridae

## Abstract

Assessments of the interplay between physiology and whole‐organism performance are fundamental to understand how individuals function in different ecological contexts. Here, we investigated the relationship between locomotor performance, androgen levels, and metabolic capacity of muscle tissues in the lizard *Tropidurus catalanensis*. We hypothesized that faster individuals would exhibit higher circulating androgen concentrations and greater metabolic capacity in skeletal and cardiac muscles, regardless of body size. We measured morphological variables, maximum sprint speed (*v*), plasma testosterone concentration, and the maximum activity of the enzymes lactate dehydrogenase (LDH) and citrate synthase (CS) in the gastrocnemius, iliofibularis, and cardiac muscles of adult males. We found that intraspecific variations in *v* were not explained by body size, plasma testosterone concentration, nor by the activity of LDH or CS in skeletal muscles. The absence of an effect of testosterone on locomotion suggests that androgen concentrations may change in response to other factors, such as environmental stressors or reproductive state. Our results indicated that the fastest lizards also had the highest CS activity in the heart. This relationship suggests that cardiac oxidative capacity plays an important role in clearing metabolites in the postexercise recovery phase. We also found a positive relationship between CS and LDH in all tissues, suggesting a functional complementarity between glycolytic and aerobic pathways that should be relevant in situations that require rapid alternation between bursts of speed and endurance, such as predator evasion or thermoregulation. Ultimately, our results highlight the importance of integrating performance and physiological traits to understand interactions between animals and their environment.

## Summary

- Intraspecific variation in sprint performance in male *Tropidurus catalanensis* was correlated with cardiac aerobic capacity.
- Aerobic and glycolytic metabolism in heart and skeletal muscle were positively associated, suggesting a mechanism of metabolic complementarity for maintaining energetic homeostasis.

## Graphical abstract

In male lizards *Tropidurus catalanensis*, sprint locomotor performance correlates with cardiac aerobic capacity.

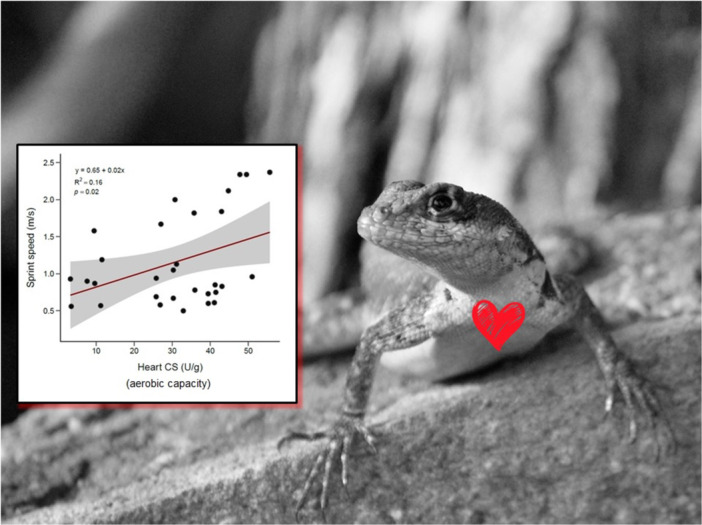

## 1 Introduction

Assessing how phenotypic traits—such as morphology, physiology, and behavior—impact individual performance is crucial to understand how organisms respond to their environment (Arnold 1983; Noble et al. 2013; Albuquerque et al. 2023). Indeed, interactions between phenotype and environment influence how efficiently organisms acquire resources, escape from predators, compete (both intra‐ and inter‐specifically), and attract mates (A. F. Bennett and Huey 1990; Cain and Ketterson 2012; Kingsolver 2003; Lailvaux and Irschick 2006; Pratt et al. 1992). The ability to move is a common denominator across different biological functions and locomotor performance is widely used as a measure of an organism's physiological capacity for movement (Irschick and Garland 2001; Kohlsdorf and Navas 2012; Wu and Seebacher 2022). Evidence suggests that individuals with larger hindlimb size, as well as greater body and muscle mass, tend to exhibit higher locomotor performance compared to smaller individuals (Garland. 1984; Huey and Hertz 1984; Kohlsdorf and Navas 2012). Indeed, this pattern has been well documented in terrestrial anurans (Citadini et al. 2018; Fabrezi et al. 2014; Rebelo and Measey 2019), lizards (Huey and Hertz 1984; Losos 1990), and birds (Barbosa and Moreno 1999; Habib and Ruff 2008; Zeffer and Norberg 2003).

Androgens like testosterone play an important role in regulating vertebrate locomotion (Hirschenhauser and Oliveira 2006; Oliveira 2004; Rusch and Angilletta 2017). For example, experimentally increasing testosterone levels enhanced sprint speed and endurance in the lizards *Sceloporus undulatus* and *Uta stansburiana* (Robson and Miles 2000; Perry et al. 2004; John‐Alder et al. 2009). Moreover, testosterone is responsible for morphological and metabolic changes that impact the performance of biologically relevant functions. Increased muscle mass (Cox, Stenquist, and Calsbeek 2009; Huyghe et al. 2009) and decreased energy reserves (Marler and Moore 1989; Marler et al. 1995) are two notable effects, since both are closely associated with success in habitat competition (Schuett 1997). Dominant individuals exhibit higher circulating testosterone levels (Rusch and Angilletta 2017) and greater muscle mass, which in turn is important for success in physical contests (Beacham 1988). Conversely, subordinate males typically exhibit escape behavior, which depends primarily on energy reserves (Pérez‐Tris et al. 2004). However, this pattern is not uniform across all lizard groups. For example, dominant adult males of the lizard *Sceloporus occidentalis* exhibited faster sprint speeds in comparison to subordinate males, regardless of muscle mass and body size (Garland et al. 1990).

Locomotor performance is also closely related to the maintenance of energy balance in muscle tissues. The metabolic capacity of an individual depends on processes such as the mobilization of phosphorylated energy compounds (e.g., phosphocreatine) in muscle fibers, as well as the coordination of anaerobic glycolysis and aerobic metabolism to support different intensities of locomotor activity (Hedrick et al. 2015). Intense and rapid activities, such as territorial defense and predator evasion, benefit from heightened anaerobic metabolism (Garland 1984), while prolonged activities like sustained vigilance and territory maintenance are supported by oxidative phosphorylation (Garland and Else 1987; Gleeson and Harrison 1988; John‐Alder 1984). Importantly, innate factors can also hamper whole‐organism performance (Garland et al. 1990). For instance, cardiac muscle performance can influence the capacity for exercise in lizards, as efficient oxygen delivery and aerobic capacity are critical for sustained physical activity (A. F. Bennett and Ruben 1979; Garland and Else 1987; Frappell et al. 2002). Evidence from the lizard *S. undulatus* suggests that intraspecific differences in endurance performance are linked to variation in cardiac performance (John‐Alder et al. 1996). However, we still have a limited understanding of the cardiovascular features that enhance oxygen delivery to support sprint locomotion in reptiles, particularly lizards.

Previous research suggested that the greater aerobic scope during intense exercise seen in monitor lizards *Varanus exanthematicus* was driven by an elevation in both stroke volume and heart rate, which led to a greater rise in cardiac output compared to a less active lizard species (Gleeson et al. 1980). Thus, increased heart capacity likely supports the high metabolic demands associated with locomotion. Furthermore, individual differences in heart mass partially accounted for intraspecific variation in endurance in the lizard *Ctenosaura similis* (Garland 1984). Even in sprint‐running lizards, which rely primarily on anaerobic metabolism to sustain locomotion, cardiac performance plays a critical role during recovery by supporting the elevated aerobic metabolic rate required to clear glycolytic byproducts and restore energy balance (Gleeson and Hancock 2002). Lizards undergo cardiovascular and respiratory adjustments following intense exercise to remove and metabolize excess lactate that is produced while running (Gleeson 1980, 1991). While these pieces of evidence support the critical role of cardiovascular traits in influencing lizard locomotor performance, our understanding of the extent to which intraspecific variation in cardiac function contributes to lizard locomotion remains limited despite its potential functional significance.

In this study, we investigated the relationship between locomotor performance and circulating androgen levels, as well as the metabolic capacity of both skeletal and cardiac muscle tissues, using adult males of Tropidurus catalanensis as a study system. This heliothermic species belongs to the *T*. *torquatus* group and has a broad distribution in southern South America (Carvalho et al. 2016). This territorial species forms a harem, engages in sustained and sprint‐based locomotion, and typically inhabits heterogeneous urban habitats (Kohlsdorf et al. 2006; Kunz and Borges‐Martins 2013; Rodrigues 1987). Evidence suggests that morphophysiological adaptations support enhanced locomotor performance in *Tropidurus* lizards, with considerable differences among individuals (Brandt et al. 2016; Kohlsdorf and Navas 2012; Kohlsdorf et al. 2006). However, while hormonal regulation and muscle metabolism have been studied independently in lizards (e.g., Gleeson and Harrison 1988; John‐Alder et al. 2009), their relative importance in facilitating locomotion, particularly in *Tropidurus*, remains poorly understood due to a lack of integrated analyses (see Kohlsdorf et al. 2004 and references therein).

Based on these pieces of information, we hypothesized that faster individuals of *T. catalanensis* would exhibit higher androgen concentrations and greater metabolic capacity in skeletal and cardiac muscles, independent of body size. Specifically, we predicted that high‐performance males (i.e., those with greater sprint speeds) would show: (1) elevated circulating androgen levels, and (2) higher activities of metabolic enzymes of glycolytic and metabolic pathways in both skeletal and cardiac muscles, even after accounting for body size variation. To this end, we measured morphological traits (body length and mass, heart mass, hindlimb length), locomotor performance (sprint speed), plasma testosterone levels, and the activity of glycolytic (lactate dehydrogenase [LDH]) and aerobic (citrate synthase [CS]) enzymes in skeletal and cardiac muscles. Through an integrative approach, our study sheds light on the relationship between whole‐organism performance and physiological activity in lizards.

## 2 Materials and Methods

### 2.1 Animal and Blood Sampling

We conducted field work between February and April of 2024, during the reproductive season of *T*. *catalanensis*, from 1400 to 1800 h, in the Parque Continental (23°32'0.18″ S; 46°45'30.47″ W) of São Paulo, Brazil. We captured 30 adult males of *T*. *catalanensis* using lassoes made of cotton string attached to fishing poles. Immediately after capture, we collected a blood sample from each individual (100 µL, within less than 2 min) via cardiac puncture using heparinized syringes (to prevent coagulation) and 22G, 1/2” needles. Blood sampling by cardiac puncture is a safe extraction method for ectothermic vertebrates (see Brown 2010; Isaza et al. 2004), including tropidurid lizards (Padilla Perez et al. 2021), as it is performed quickly and poses minimal risk to the animals. The likelihood of collection errors is low, and hemorrhaging is easily controlled due to the relatively low intraventricular pressure in the vascular system compared to endothermic vertebrates (Isaza et al. 2004). We marked each individual with permanent markers, labeled the corresponding blood samples, and stored the samples on ice until further processing. We centrifuged each sample to separate and extract the plasma (CT‐6000 Craltech, Brazil, 1000 g, 4 min), which we stored in cryotubes and preserved frozen in a commercial −20°C freezer until transportation to the laboratory, where the samples were stored in a −80°C freezer.

At the time of capture, we also recorded body temperature (*T*_b_), air temperature (*T*_air_), substrate temperature (*T*_sub_), and snout‐vent length (SVL). We used a fast‐response thermometer (DeltaTrak model #11002, nearest 0.1°C) to measure *T*_b_ by inserting the probe 2 cm into the cloaca within 10 s of capture, *T*_air_ at 1 m above the substrate (in the shade), and *T*_sub_ directly in contact with the substrate. We measured SVL with a ruler (nearest 0.1 cm). Then, we placed the lizards individually in breathable bags to ensure proper ventilation during transportation to the Laboratory of Ecology, Zoology, and Comparative Physiology at The Federal University of São Paulo (UNIFESP). We performed all protocols after authorization of the Ethics Committee on Animal Use (CEUA/UNIFESP, no. 8086091023). Our sampling and field work was authorized by the Biodiversity Authorization and Information System (SISBio/Brazil, no. 229124).

### 2.2 Husbandry

In the laboratory, we housed up to four lizards in plastic terraria (40 cm L × 20 cm W × 30 cm H) with perforated lids to ensure proper ventilation. We equipped the lid of each container with a 70 W halogen lamp as a heat source, positioned approximately 20 cm above the substrate to prevent direct contact with the animals and allow for behavioral thermoregulation (temperature in the terraria ranged from 20°C to 50°C). Terraria had cardboard substrate and concrete blocks to be used as shelters or as sites for thermoregulation. We fed the lizards with crickets and small mealworms upon capture, while water was available ad libitum through the experiments. We fasted the lizards 2 days before the experiments to avoid postprandial effects and to ensure a postabsorptive state.

### 2.3 Testosterone

We used plasma testosterone concentration (expressed in ng/mL) as a measure of androgen levels. We processed plasma samples following the methods described by Assis et al. (2019). Briefly, we added 5 mL of ether to 10 µL of each sample, followed by vortex agitation for 30 s and centrifugation (CT‐6000 Craltech, Brazil, 4°C, 9 min, 1000 *g*). We then cooled the samples at −80°C for 7 min to allow for phase separation, after which we transferred the liquid phase to a new tube. We placed these tubes in a fume hood at room temperature (24 ± 2°C) until the ether completely evaporated (approximately 48 h). Subsequently, we resuspended the samples in enzyme immunoassay (EIA) buffer, and we measured testosterone concentration using commercially available EIA kits (Arbor Assays, Ann Arbor, MI, USA), following the manufacturer's protocol (kit #K032‐H1/H5).

### 2.4 Locomotor Performance

Although male *T. catalanensis* lizards are capable of sustained locomotion in the field, their movement primarily relies on repeated sprint performances. We therefore quantified maximum speed (*v*) on a 1.5 m long horizontal surface as a measure of sprint speed (Kohlsdorf et al. 2004; Kohlsdorf and Navas 2012; Brandt et al. 2016). We conducted all measurements within 1 week of capture to minimize potential acclimation effects induced by captivity. On the day of the trials, we kept the individuals in a climate chamber (Eletrolab Ltda., Brazil) at 35 ± 1.5°C for 30 min to allow for thermal equilibrium at their preferred temperature (see Kohlsdorf et al. 2006; Padilla Perez et al. 2021). After thermal equilibration, we transferred the individual to the running track (200 cm L × 8.5 W × 9 cm H; made from transparent glass). The track had designated “start” and “finish” lines, resulting in an effective running distance of 1.5 m (Brandt et al. 2016). We used sandpaper as a substrate, and we manually stimulated the lizard to run using gentle tail taps. We recorded each run with a Poco X6 Pro (Xiaomi, China) at 120 frames per second, ensuring precise determination of start and finish times with a temporal resolution of 8.333 ms per frame. We positioned the camera perpendicularly to the running track, at a height of 1.5 m, allowing for adequate framing of the entire racetrack in the video. We measured running time (*t*) from the recordings using DaVinci Resolve Studio software (version 18.6.5, build 7, Blackmagic Design Pty. Ltd., USA), considering the time elapsed between the moment the individual crossed the start line and the moment it crossed the finish line. Then, we calculated maximum sprint speed (m/s) by dividing the total distance covered (1.5 m) by the total running time (in seconds). We performed these measurements on two different days (minimum interval of 24 h between trials), providing two measurements per individual. We selected the highest value between the two trials. We also measured body temperature after each run (*T*_br_, using the same protocol for *T*_b_ measurements in the field) and, after the last run, we measured body mass (M_b_) with an analytical scale (AUY220, Shimadzu Corp., USA; nearest 0.0001 g), as well as femur length (FEL), tibia length (TL), and foot length (FOL) of the left hindlimb for each individual using a caliper (nearest 0.01 mm).

### 2.5 Enzyme Activity

After *v* measurements, we euthanized each individual with an intraperitoneal overdose of thiopental (> 100 mg**/**kg). Then, we dissected the gastrocnemius and the iliofibularis muscles of each individual's left leg. The gastrocnemius muscle functions as a flexor in the lower leg, pulling the foot backward and propelling the body forward during movement. It is activated during the swing phase of running or walking when the ankle is extended, and the knee is flexed (Higham et al. 2011). Meanwhile, the iliofibularis muscle flexes the upper leg, pulling the hind leg backward and moving the body in the direction of locomotion. This muscle is also active during the swing phase of running or walking when the femur is abducted, and the knee is bent (Jayne et al. 1990). We also excised the entire cardiac ventricle by severing the great vessels and atria (Jensen et al. 2014), and measured its mass (*M*_h_) using a precision scale (AUY220, Shimadzu Corp., USA; nearest 0.0001 g). We stored all tissues in a −80°C ultra freezer (MDF‐U53VC, Sanyo Sci., Japan).

From these muscle tissue samples, we measured the activity of the enzymes LDH and CS as proxies for glycolytic and aerobic metabolic capacities, respectively. For enzyme activity measurements, we homogenized each sample on ice in nine volumes of Imidazole‐HCl 20 mmol L^−1^ buffer (pH 7.4) containing ethylenediaminetetraacetic acid 2 mmol L^−1^, NaF 20 mmol L^−1^, phenylmethylsulfonyl fluoride 1 mmol L,^−1^ and Triton X‐100 0.1% using a Turrax‐type homogenizer (Ultra Stirrer 80, Eikonal ltda., Brazil). Subsequently, we disrupted mitochondrial membranes via sonication using a Misonix XL2000 sonicator (Qsonica L.L.C., Connecticut, USA) in three 10 s intervals, to yield the homogenate. For all enzyme assays, we used reaction media as described in Bergmeyer (1983). For LDH (E.C.1.1.1.27): 100 mmol L^−1^ imidazole (pH 7.0), 5 mmol L^−1^ dithiothreitol, 0.15 mmol L^−1^ NADH, 1:30 diluted homogenate, and 1 mmol L^−1^ pyruvate (omitted in the control assay); for CS (E.C. 4.1.3.7): 50 mmol L^−1^ Trizma (pH 8.0), 0.1 mmol L^−1^ DTNB, 0.2 mmol L^−1^ acetyl‐CoA, 1:10 diluted homogenate and 0.9 mmol L^−1^ oxaloacetate (omitted in the control assay). We assessed enzyme activity based on absorbance changes of the reduced form of the nicotinamide adenine dinucleotide (NADH) at 340 nm (for LDH) and 5,5’‐dithiobis‐2‐nitrobenzoic acid (DTNB) at 412 nm (for CS), under substrate saturation and non‐inhibitory conditions, following modifications from Bergmeyer (1983). We performed all measurements at 35°C (the same temperature used for *v* trials) in quartz cuvettes in a final volume of 700 μL, using a Beckman DU‐800 spectrophotometer equipped with a Peltier temperature controller (Beckman Inc., USA). We calculated enzyme activity by multiplying the changes in absorbance per minute after substrate addition (minus the control absorbance variation, without substrate) by the total sample dilution, and then dividing by the molar absorption coefficient (mL/mol cm) multiplied by the path length of the light beam in the solution (1 cm for all assays). The molar absorption coefficient values were 6.22 for LDH and 13.6 for CS. We performed all assays in duplicates and expressed the enzyme activity as μmol of substrate converted into product per minute per gram of wet tissue mass (μmol/min g wet mass).

### 2.6 Statistical Analyses

We performed all analyses using R (version 4.4.1) in RStudio (version 2024‐06‐14) with a significance level of 0.05. Before analyses, we log10‐transformed all morphological variables. We assessed the relationship between *T*_sub_ and *T*_air_ with the Pearson correlation coefficient fit through the “cor.test” function from the stats package (R Core Team 2024). To test the relationship among body size variables, we built a generalized linear model (GLM) with the “glm” function from the stats package (R Core Team 2024). This model had SVL as the predictor, and *M*_b_, *M*_h_, FEL, TL, and FOL as response variables. To evaluate how the ratio of CS to LDH differed among tissues, we built a linear mixed‐effects model (LMM) using the “lmer” function from the lme4 package (Bates et al. 2015). This model had the CS/LDH ratio as the response variable, tissue type (gastrocnemius, iliofibularis, and heart) as the predictor variable, and ID as a random term to account for repeated measures. To test the hypothesis that locomotor performance would vary as a function of androgen levels and enzyme activity, we built a GLM that had *v* as the response variable, and testosterone level, CS activity, LDH activity, and log‐transformed SVL as predictor variables. We fit one model per tissue type, and then we used the “dredge” function from the MuMIn package (Bartoń 2020) to rank the best model based on the Akaike Information Criterion corrected for small sample sizes (AICc) (Anderson and Burnham (2002). For each tissue, we also evaluated the relationship between CS and LDH activity through the “cor.test” function from the stats package (R Core Team 2024). For each model, we assessed residual autocorrelation with the “checkresiduals” function from the forecast package (Hyndman et al. 2020), and the “acf” and “qqnorm” functions from the stats package (R Core Team 2024). We created figures using the ggplot2 package (Wickham 2016).

## 3 Results

**Figure 1:**
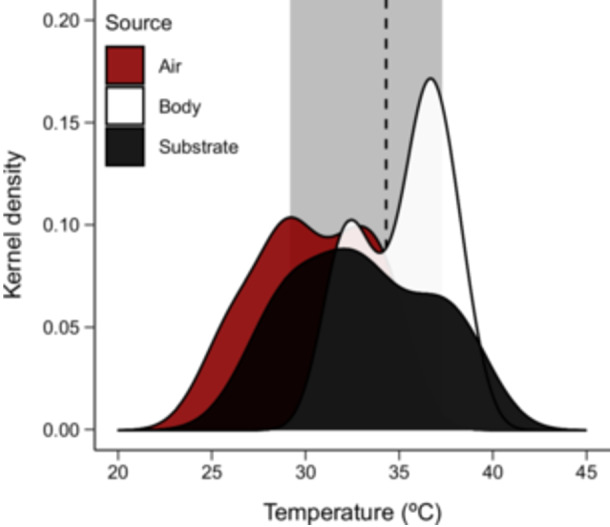
Kernel density estimates of thermal ecology parameters of male *Tropidurus catalanensis* (*N* = 30). The shaded area shows the range of preferred body temperatures, and the vertical dashed line shows the mean preferred body temperature for our study population based on Padilla Perez et al. (2021).

We found that *T*_sub_ and *T*_air_ were positively correlated in the area where we sampled our individuals of *T. catalanensis* (Pearson's *r* = 0.57, *p* < 0.001), with *T*_air_ = 30.39 ± 3.04°C and *T*_sub_ = 32.98 ± 3.70°C (mean ± standard deviation). In the field, male *T*. *catalanensis* sustained a mean *T*_b_ of 35.24 ± 2.32°C (Figure 1). Body mass was positively associated with SVL (GLM, *β* = 0.20, *t* = 4.02, *p *< 0.001, Figure S1) (see Table 1 for descriptive analysis), while no other morphological variables showed associations with SVL (Table S1).

**Table 1:** Summary of morphological variables for male *Tropidurus catalanensis*.

	*N* = 30
*M*_b_ (g)	
Mean ± SD	40.62 ± 10.97
Median (min, max)	41.10 (20.80, 64.70)
*M*_h_ (g)	
Mean ± SD	0.09 ± 0.03
Median (min, max)	0.08 (0.04, 0.16)
SVL (cm)	
Mean ± SD	10.68 ± 0.94
Median (min, max)	10.70 (9.10, 12.70)
Femur length (cm)	
Mean ± SD	2.70 ± 0.23
Median (min, max)	2.71 (2.26, 3.07)
Tibia length (cm)	
Mean ± SD	2.67 ± 0.19
Median (min, max)	2.68 (2.21, 2.99)
Foot length (cm)	
Mean ± SD	3.31 ± 0.21
Median (min, max)	3.36 (2.89, 3.67)

Abbreviations: M_b_ = body mass, M_h_ = heart mass, Max = maximum value, Min = minimum value, SD = standard deviation, SVL = snout‐vent length.

**Figure 2:**
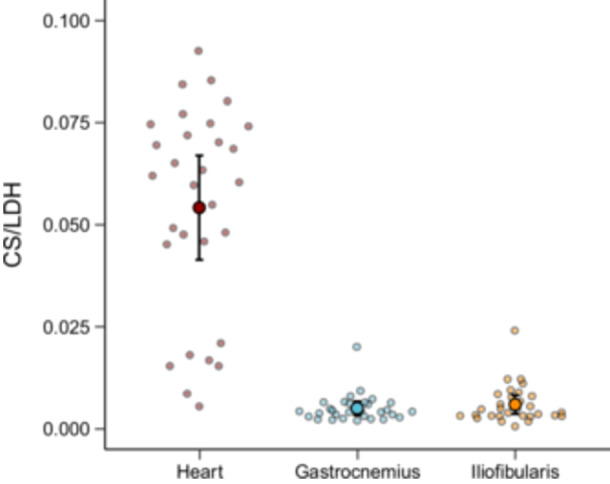
Citrate synthase (CS) to lactate dehydrogenase (LDH) ratio among heart and skeletal (gastrocnemius and iliofibularis) muscles of male *Tropidurus catalanensis* (*N* = 30). Large dots show mean ratio values, and the error bars represent the 95% confidence interval of the mean. Small dots show individual ratio values across tissue types.

During the locomotor performance trials, mean lizard *T*_br_ was 32.97 ± 1.35°C and *v *= 1.16 ± 0.61 m/s (range: 0.5–2.37 m/s). CS activity were pronounced higher in heart muscle (descriptive analysis in Table 2). The CS/LDH ratio differed among tissues (LMM, *χ*²_[2]_ = 209.88, *p *< 0.001), being lower in the skeletal muscles (gastrocnemius and iliofibularis) than in the heart muscle (Figure 2). Plasma testosterone concentration ranged from 0.17 to 60.36 ng/mL and averaged 7.71 ± 12.44 ng/mL.

**Table 2:** Activity of citrate synthase (CS) and lactate dehydrogenase (LDH) in skeletal (gastrocnemius and iliofibularis) and heart muscles of male *Tropidurus catalanensis* (*N* = 30).

	Gastrocnemius		Iliofibularis		Heart	
	CS	LDH	CS	LDH	CS	LDH
Activity (U/g)						
Mean ± SD	3.23 ± 1.81	639.12 ± 214.31	3.87 ± 2.44	688.24 ± 247.73	30.87 ± 14.94	574.33 ± 118.65
Median (min, max)	2.93 (0.58, 7.17)	696.62 (224.68, 946.29)	3.25 (0.33, 11.18)	738.35 (205.20, 1120.30)	32.02 (3.26, 55.63)	574.18 (360.29, 819.50)

Abbreviation: SD = standard deviation.

**Figure 3:**
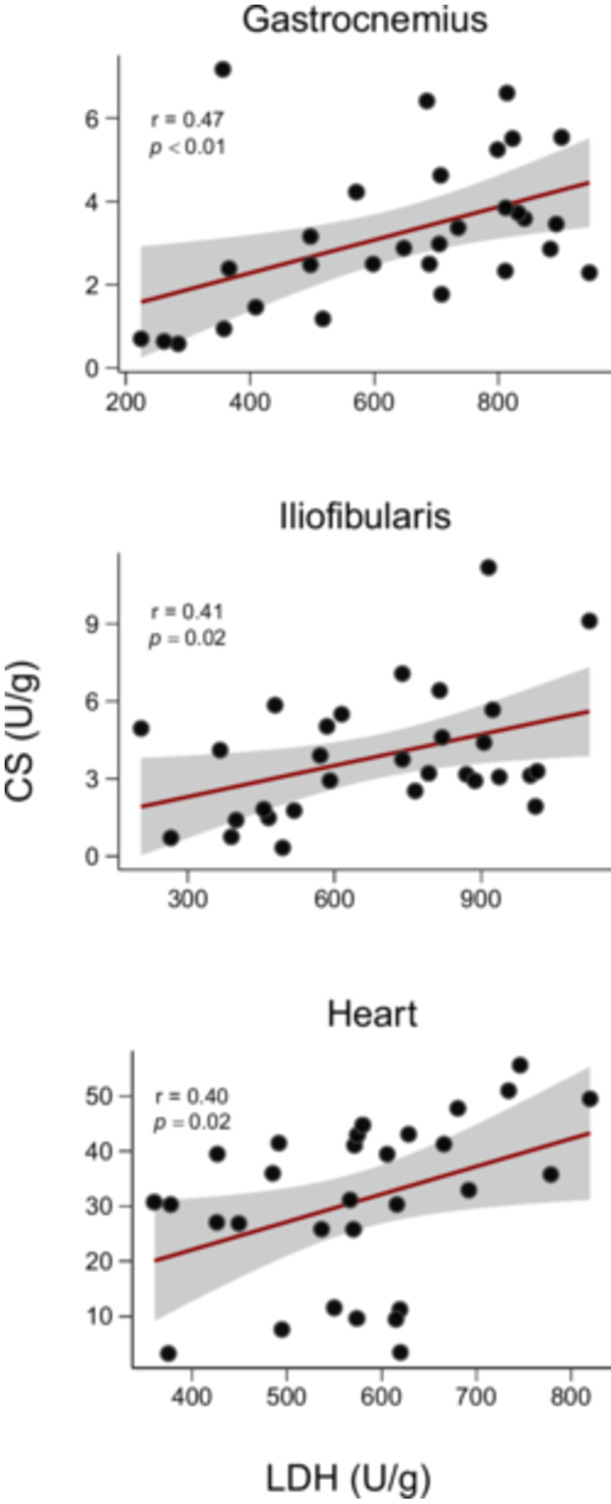
The relationship between citrate synthase (CS) and lactate dehydrogenase (LDH) activities in the skeletal (gastrocnemius and iliofibularis) and heart muscles of male *Tropidurus catalanensis* (*N* = 30). The solid red line shows the predicted relationship between the two variables, and the gray shaded area shows the 95% confidence interval. Black dots represent individual values.

**Figure 4:**
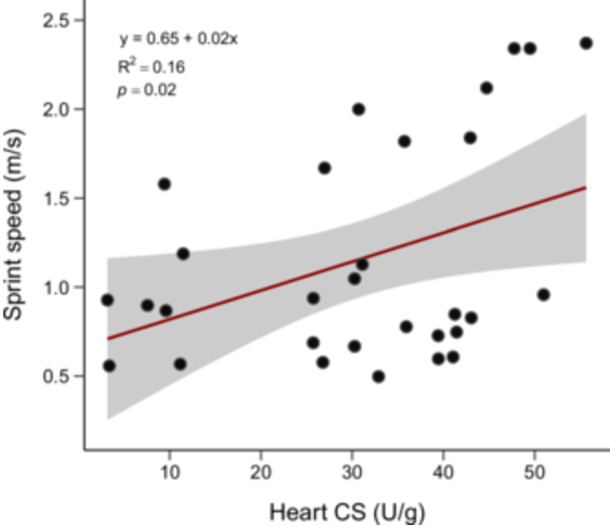
The relationship between maximum sprint speed (*v*) and citrate synthase (CS) activity in the heart muscle of males *Tropidurus catalanensis* (*N* = 30). The solid red line shows the predicted relationship between the two variables, and the gray shaded area shows the 95% confidence interval. Black dots represent individual values.

**Figure 5:**
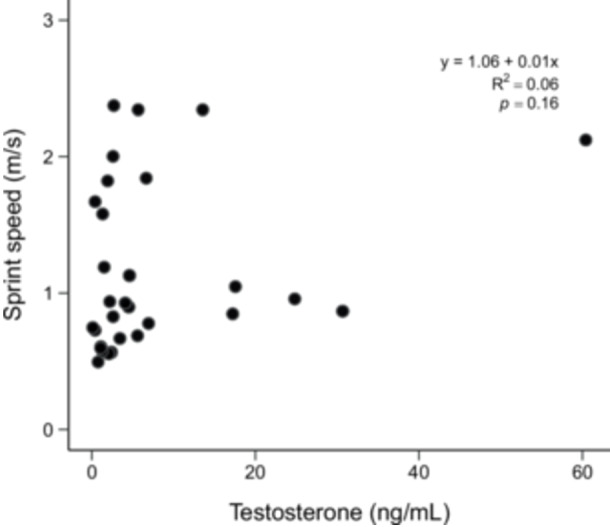
Maximum sprint speed (*v*) as a function of plasma testosterone levels in males *Tropidurus catalanensis* (*N* = 30). Black dots represent individual values.

In all tissues, CS was positively correlated with LDH (Pearson's, gastrocnemius: *r* = 0.47, *p* = 0.008; iliofibularis: *r* = 0.41, *p* = 0.02; heart: *r* = 0.40, *p *= 0.02) (Figure 3). Model selection based on AICc (weight = 0.22) indicated that activity of CS in the heart best explained the observed variation in *v* (GLM, *β* = 0.16, *t* = 2.28, *p *= 0.02), with no contribution of body size (Table 3, Figure 4). Sprint speed was not associated with plasma testosterone levels (Figure 5).

**Table 3:** Model selection based on the Akaike Information Criterion corrected for small sample sizes (AICc).

Intercept	CS_h_	LDH_h_	SVL	Testosterone	df	loglikelihood	AICc	Δ AICc	*w*
0.658	0.016	—	—	—	3	−24.64	56.2	0.00	0.22
−2.375	0.014	—	1.307	—	4	−24.019	57.6	1.43	0.10
0.632	0.015	—	—	0.009	4	−24.035	57.7	1.47	0.10
−11.330	—	0.990	2.630	—	4	−24.138	57.9	1.67	0.09
−1.009	0.014	0.270	—	—	4	−24.499	58.6	2.40	0.067
1.159	—	—	—	—	2	−27.212	58.9	2.66	0.059
−3.282	—	—	1.878	—	3	−26.046	59.0	2.81	0.054
−7.935	0.009	0.649	1.976	—	5	−23.328	59.2	2.95	0.051
1.063	—	—	—	0.012	3	−26.213	59.3	3.15	0.046
−2.965	—	0.651	—	—	3	−26.396	59.7	3.51	0.038
−1.728	0.014	—	1.019	0.007	5	−23.677	59.9	3.65	0.036
−0.931	0.013	0.254	—	0.008	5	−23.906	60.3	4.11	0.028
−10.210	—	0.920	2.327	0.005	5	−23.934	60.4	4.16	0.028
−2.644	—	0.586	—	0.011	4	−25.515	60.6	4.43	0.024
−2.390	—	—	1.471	0.009	4	−25.537	60.7	4.47	0.024
−6.884	0.01	0.584	1.689	0.005	6	−23.127	61.9	5.70	0.013

*Note:* This model considered maximum sprint speed (*v*) as the response variable, and citrate synthase activity in the heart (CS_h_), lactate dehydrogenase activity in the heart (LDH_h_), log‐transformed snout‐vent length (SVL), and testosterone levels as predictors.

## 4 Discussion

In this study, we employed an integrative approach to evaluate the relationship between locomotor performance, circulating androgen levels, and muscle metabolic activity in *T*. *catalanensis*. We hypothesized that males with higher sprint speeds would exhibit (1) increased circulating androgen levels, and (2) greater activity of glycolytic and oxidative metabolic enzymes in both skeletal and cardiac muscles, even after controlling for body size effects. We found that individuals varied widely in terms of locomotor performance at an ecologically relevant body temperature, although such variation was independent of body size, plasma testosterone concentration, or enzymatic activity in skeletal tissues. Furthermore, our data demonstrated that faster individuals exhibited greater cardiac aerobic capacity, suggesting that interindividual variation in locomotor performance is partially mediated by heart metabolic characteristics. Under natural conditions, the body temperature of our sample of *T. catalanensis* matched the preferred temperature range previously established for this population (Padilla Pérez et al. 2021). While femur and tibia length play an essential role during the final propulsion phase of running by increasing speed (Fieler and Jayne 1998; Garland and Losos 1994; Reilly and Delancey 1997; Spezzano and Jayne 2004), our results support the proposition that locomotor performance on horizontal surfaces is independent of morphology (see similar conclusion in Garland 1984). This finding complements previous research demonstrating that hindlimb size may be more important for climbing performance in *T*. *catalanensis* (Brandt et al. 2016). Thus, the extent to which morphology may influence whole‐organism performance may differ between modes of locomotion. Sprint locomotion on horizontal surfaces may therefore be determined by other factors, such as the composition and proportion of leg muscle fibers, which influence skeletal muscle contractile capacity and the capacity for adenosine triphosphate (ATP) synthesis via glycolytic or aerobic pathways (Gleeson and Harrison 1988; Kohlsdorf and Navas 2012).

We did not find support for the hypothesis that the fastest lizards would show the highest circulating testosterone levels. The available literature on androgen effects on locomotion is mixed. Some studies suggested a direct effect of testosterone levels on locomotor performance (Noble et al. 2014) while others found that testosterone had no effect on locomotion (Cox, Stenquist, Henningsen, et al. 2009; Cox, Stenquist, and Calsbeek 2009; Huyghe et al. 2009; Johnson et al. 2011; Kubička et al. 2015). Our work lends support to the latter group of studies. In fact, testosterone has a broad effect in several behavioral, physiological and morphological characteristics of lizards (Sinervo et al. 2000), but most of the effects seem to be pleiotropic to coordinate the expression of diverse phenotypic traits (Cox, Stenquist, Henningsen, et al. 2009). Thus, the ability to determine a direct correlation between testosterone and locomotion might be contingent on other factors such as social dominance among individuals (Perry et al. 2004). It is worth highlighting that our results were obtained from lizards directly sourced from nature, and that we do not dispute the evidence showing that experimental manipulation of androgen levels enhances sprint speed (John‐Alder et al. 2009). However, androgens may mediate other physical activities in reptiles besides locomotion, especially during the breeding season (John‐Alder et al. 2009). For instance, testosterone has been shown to influence bite force in breeding lizards, which is the period in which androgen levels are at their peak (Gowan et al. 2010; Huyghe et al. 2009). Alternatively, our experiments may have taken place outside of a critical window in which androgen effects on locomotion may be captured. To clarify how androgen levels regulate locomotion, future studies may compare how testosterone levels and *v* measured at the peak of the reproductive season (spring–summer) differ from measurements during the provisioning season (autumn) (see also Angilletta et al. 2002; Lailvaux et al. 2003; Husak 2006; Gowan et al. 2010).

As expected, we found a predominance of glycolytic capacity in relation to aerobic ATP synthetic pathways in both skeletal muscles of *T. catalanensis*, as evidenced by the low CS/LDH ratios in the gastrocnemius and iliofibularis muscles. This is consistent with the rapid escape behavior characteristic of many lizards and jumping amphibians, wherein explosive performance is primarily supported by phosphocreatine and by catabolism of glucose into lactate (Garland. 1984; Garland and Else 1987; Pough and Andrews 1985; Kohlsdorf et al. 2004). In *T. catalanensis*, however, sprint speed at an ecologically relevant body temperature was not explained by enzyme activity in skeletal muscles; a finding that runs counter to our hypothesis. The comparatively higher CS/LDH ratio in the heart reflects the aerobic nature of cardiac muscle fibers, which is further reinforced by the fact that the LDH isoform in heart muscle preferentially catalyzes the lactate‐to‐pyruvate conversion (Hochachka and Somero 2002) to further support aerobic metabolism.

Our findings partially support the hypothesis that sprint speed would be correlated with enzyme activity in skeletal and heart tissues. Specifically, we found that the fastest lizards were the ones with higher CS activity in the heart muscle. Sustained locomotor activity in several lizard species is associated with elevated cardiac output to enhance oxygen delivery to active skeletal muscles during exercise (A. Bennett 1974; Hedrick et al. 2015; Albuquerque et al. 2023). However, given its brief time‐course and the highly glycolytic nature of the muscles used during sprinting, it is unlikely that cardiac function is directly related to sprint performance. We suggest that this relationship is possibly driven by two non‐mutually exclusive possibilities. First, cardiac oxidative capacity plays a crucial role in the postexercise recovery phase, ensuring metabolite (e.g., lactate) clearance through oxidation (Gleeson and Bennett 1982; Gleeson and Hancock 2002). This process facilitates the restoration of metabolic and energetic balance, reducing the time of recovery and preparing the organism for future locomotor challenges. Previous research demonstrated that larger *T*. *catalanensis* with proportionally larger hearts were capable of running longer distances (Maia‐Carneiro et al. 2021). Based on our findings, one may also infer that heart function should aid in postexercise recovery in this species. Second, we acknowledge that cardiac capacity may be related to territorial defense, mating contests or other sustained activities. These activities are expected to be correlated more with endurance than power or sprint speed. However, individuals with enhanced capacity for performance in a given task may also possess enhanced capacity to perform other tasks. For example, individuals that sustain relatively high baseline energetic costs also expend more energy when performing high‐intensity activities (Auer et al. 2017). In support of this pattern, somewhat surprisingly, we also found a positive correlation between CS and LDH activity in all the tissues considered here, suggesting a lack of trade‐offs between oxidative and glycolytic fiber types. In skeletal muscles, this positive correlation between CS and LDH suggests a functional complementarity between glycolytic and aerobic pathways. This finding runs counter to the trade‐off between power and endurance seen in the majority of studies to date (e.g., A. F. Bennett et al. 1984; Herrel and Bonneaud 2012; Huey, Bennett, et al. 1984; Vanhooydonck et al. 2001; Wilson et al. 2002), as was also shown for different species of lizards (Vanhooydonck et al. 2014). Nonetheless, in heliothermic lizards, such metabolic flexibility may be crucial in situations that require rapid alternation between bursts of speed and endurance, such as predator evasion or behavioral thermoregulation (Giacometti et al. 2024).

In the heart ventricle muscle, the positive correlation between CS and LDH suggests that, although aerobic metabolism predominates for efficient and continuous energy supply, LDH isoform catalysis may play a supportive role under conditions of high energetic demand by converting lactate into pyruvate, thereby promoting substrate entry into the mitochondria for aerobic ATP production (Wang et al. 2018; Hochachka and Somero 2002). In turn, this process should allow the heart to maintain its essential function of generating vascular pressure even under adverse conditions, ultimately ensuring adequate oxygen delivery and nutrient transport to peripheral tissues (Gleeson et al. 1980). Together, our complementary findings indicate that *T. catalanensis* exhibits a coordinated relationship between aerobic and anaerobic metabolic capacities across different tissues, which assists the maintenance of a homeostatic state while lizards perform energetically demanding functions such as high‐speed locomotion.

## 5 Conclusions

In *T*. *catalanensis*, intraspecific differences in locomotor performance were not explained by differences in morphology, plasma testosterone levels, or enzyme activity in skeletal muscles. Instead, we found that the fastest lizards were the ones with greater cardiac aerobic capacity. This finding identifies an important relationship between cardiac aerobic metabolism and sprint speed and aligns with previous research suggesting enhanced aerobic capacity in *T*. *catalanensis* relative to congeners (Giacometti et al. 2022). We suggest that during sprint‐running—a process which is sustained primarily by anaerobic metabolism—cardiac performance plays an essential role in postexercise recovery by maintaining the increased aerobic metabolic rate that is necessary to eliminate glycolytic byproducts and reestablish energetic homeostasis. Beyond that, we also found aerobic and glycolytic metabolic capacities to be positively related across skeletal and cardiac tissues in *T. catalanensis*. Particularly in the heart, the role played by LDH isoform in converting lactate to pyruvate can contribute to the aerobic nature of ATP production to sustain energetic homeostasis. We still need further investigation about the apparent lack of a trade‐off between aerobic and glycolytic metabolism, however. Nevertheless, this metabolic integration possibly provides functional flexibility, thereby allowing organisms to meet the energetic demands of different biological functions. Ultimately, our results demonstrate that integrating organismal performance data with physiological measurements provides critical insights into lizard functional biology.

## Conflicts of Interest

The authors declare no conflicts of interest.

## Supporting information

Figure S1_Lima et al.

Supporting Information_Tropidurus catalanensis_Lima et al_R1.

data set_Lima et al.

## Data Availability

Our data can be accessed from Zenodo: https://doi.org/10.5281/zenodo.16814971.
