# Supplementary figures and images for: Closer to the Heart: Cardiac Muscle Aerobic Capacity Correlates With Intraspecific Variation in Sprint Performance Rather Than Androgen Levels in the Neotropical Lizard Tropidurus catalanensis
Source: J Exp Zool A Ecol Integr Physiol. 2025 Sep 22;345(1):16–26. doi: 10.1002/jez.70040 (PMC12705342; doi:10.1002/jez.70040)

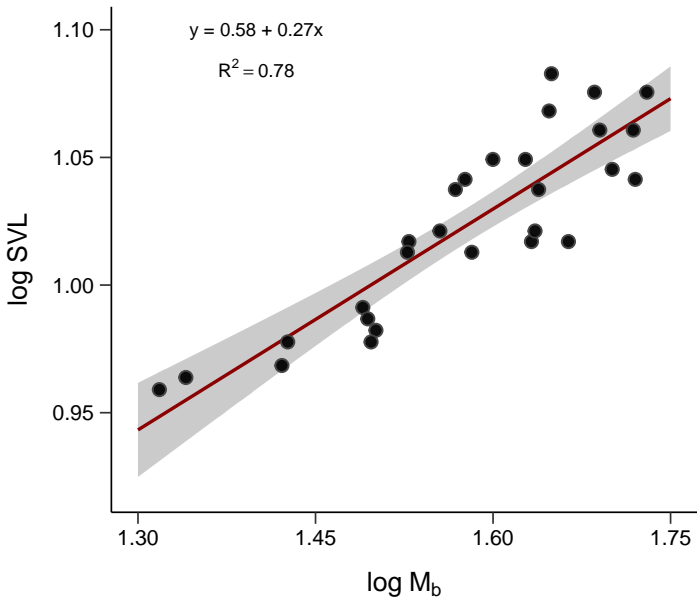

Supplement: Supplementary file 1 — Figure S1_Lima et al. [file JEZ-345-16-s001.pdf]
