# Supplementary material for: Closer to the Heart: Cardiac Muscle Aerobic Capacity Correlates With Intraspecific Variation in Sprint Performance Rather Than Androgen Levels in the Neotropical Lizard Tropidurus catalanensis
Source: J Exp Zool A Ecol Integr Physiol. 2025 Sep 22;345(1):16–26. doi: 10.1002/jez.70040 (PMC12705342; doi:10.1002/jez.70040)
Supplement: Supplementary file 2 — Supporting Information_Tropidurus catalanensis_Lima et al_R1. [file JEZ-345-16-s002.docx]

**Closer to the heart: cardiac muscle aerobic capacity correlates with intraspecific variation sprint performance rather than androgens in the neotropical lizard Tropidurus catalanensis**

Willian Souza Lima, Danilo Giacometti, Paul J. Schaeffer, José Eduardo de Carvalho

**Supporting Information**

**Table S1.** Parameter estimates (β), 95% confidence intervals (95% CI), and *p*-values for the model assessing the relationship between log-transformed snout-vent length (SVL), and log-transformed body mass (M_b_), heart mass (M_h_), femur length (FL), tibia length (TL), and foot length (FtL) in *Tropidurus catalanensis* (N = 30). Significant parameters are shown in bold.

|  | **logSVL ~ logM_b_ + logM_h_ + logFL + logTL + logFtL** | | |
| --- | --- | --- | --- |
| *Predictors* | *Estimates* | *95% CI* | *p* |
| Intercept | 0.63 | 0.41 – 0.85 | **<0.001** |
| logMb | 0.20 | 0.10 – 0.29 | **<0.001** |
| logMh | 0.05 | -0.02 – 0.12 | 0.179 |
| logFL | 0.02 | -0.33 – 0.37 | 0.902 |
| logTL | 0.08 | -0.33 – 0.49 | 0.705 |
| logFtL | 0.18 | -0.17 – 0.53 | 0.310 |
| Observations | 30 | | |
| R^2^ | 0.826 | | |

**Figure S1.** Allometric relationship between log10-transformed snout-vent length (SVL) and body mass (M_b_) of males *Tropidurus catalanesis* (N =30). The solid red line shows the relationship between the two variables, and the grey shaded area shows the 95% confidence interval. Black dots represent individual values.
